# Application of calcifying bacteria for remediation of stones and cultural heritages

**DOI:** 10.3389/fmicb.2014.00304

**Published:** 2014-06-26

**Authors:** Navdeep Kaur Dhami, M. Sudhakara Reddy, Abhijit Mukherjee

**Affiliations:** ^1^Department of Biotechnology, Thapar UniversityPatiala, India; ^2^Department of Civil Engineering, Curtin UniversityPerth, WA, Australia

**Keywords:** limestone, microbial carbonates, bacteria, urease, biofilm, extrapolymeric substances, calcite

## Abstract

Since ages, architects and artists worldwide have focused on usage of durable stones as marble and limestone for construction of beautiful and magnificent historic monuments as European Cathedrals, Roman, and Greek temples, Taj Mahal etc. But survival of these irreplaceable cultural and historical assets is in question these days due to their degradation and deterioration caused by number of biotic and abiotic factors. These causative agents have affected not only the esthetic appearance of these structures, but also lead to deterioration of their strength and durability. The present review emphasizes about different causative agents leading to deterioration and application of microbially induced calcium carbonate precipitation as a novel and potential technology for dealing with these problems. The study also sheds light on benefits of microbial carbonate binders over the traditional agents and future directions.

## Introduction

Taking a look at our history, beautiful monuments and sculptures of limestone are seen. Be it European Cathedrals (Milan Cathedral, Italy), Roman and Greek temples, the Taj Mahal and the Pyramids, limestone is everywhere. Numerous limestone and marble quarries encouraged ancient Greek architects to build the Acropolis and Roman architects to build magnificent Forum (Corvo et al., 2000). Limestone, consisting almost entirely of calcite (the most stable polymorph of calcium carbonate) along with a small content of aragonite has been found to be highly durable building material since ages (Graedel, 2000). Though known for its great strength, these materials are highly porous and hydrophilic in nature making them highly susceptible to water (such as acid rain) and environmental pollutants (Tiano et al., 1999). The water carrying harmful and corrosive ions often penetrates into the pores of these stones leading to their deterioration. Architectural and sculptural stones have undergone deterioration due to number of factors such as physical, chemical, and biological weathering (Rodriguez-Navarro and Sebastian, 1996; Wakefield and Jones, 1998; Rodriguez-Navarro and Doehne, 1999). Several environmental pollutants, particulate matters, fly ash, smog and natural causes lead to degradation of these structures (Fernandes, 2006; Chand and Cameotra, 2011). Microorganisms inhabiting these structures, upon interaction with other detrimental biotic and abiotic factors, also promote weathering and corrosion of these building materials (Fernandes, 2006). All these factors are thereby causing great harm to physical and esthetic appearance of such structures. Because of all these reasons, survival of many cultural and historical assets is in threat. One of such examples is the cave of Lascaux in southwest France where infection of *Fusarium* sp. and other molds deteriorated the floor and banks of main chamber (Rosenbaum, 2006). Paintings in Altamira cave in Santillana del Mar, Spain, and the earliest known Christian paintings adorning Roman catacomb walls have also undergone similar fate.

The problem of deterioration of these historical monuments has fetched the attention of archeologists, geobiologists and bioconservators to preventive and remedial technologies to safeguard these cultural heritage monuments, which is a big challenging task. Many attempts have been made to remediate such structures by application of conservative treatments using organic and inorganic products (Lazzarini and Laurenzi Tabasso, 1986), but these agents have not been as effective as expected due to complex nature of the textures/compositions of materials encountered. Other drawback is that high amounts of organic solvents are often wasted in the environment and in few cases, these treatments even led to detrimental effects on the stone material in context to its texture, physical strength and esthetic appearance (González-Muñoz, 2008). None of the tested conventional treatment methods have proved to be satisfactory for the preservation and consolidation of these deteriorated monuments (Cappitelli et al., 2007; González-Muñoz, 2008) (Table 1). Hence, the durability related issues are causing high impact on national economies as huge sums of money are required for maintenance and repair of such structures. The short comings of conventional methods have encouraged the search and development of new conservation treatments for remediation and protection of these magnificent materials, based on biological methods (Fernandes, 2006).

**Table 1:** **Methodologies for eradication of degradative agents of stone works**.

**Method**	**Advantages**	**Disadvantages**	**References**
**PHYSICAL METHODS**			
UV, Gamma, X irradiation	Simple, high penetration of gamma and X, effective on insects, UV effective on microbes	Application in movable or small scale objects, poor penetration of UV	Warscheid and Braams, 2000; Salvadori, 2003
Mechanical removal of biological material by hand or tool	Traditional and widely used	Short lived results, only superficial mycelium removed, microbes redevelop, damage stone	Dakal and Cameotra, 2012
Low pressure water rinsing/ steam cleansing	Effective for removal of algae, mosses, lichens, no health hazards	Water retained in pores likely to favor microbial growth	Kumar and Kumar, 1999
**CHEMICAL METHODS**			
Nongaseous biocides	Broad and narrow spectrum	Health hazards, unwanted side effects, inadequate timing of application	Kumar and Kumar, 1999; Salvadori, 2003; Cappitelli and Sorlini, 2005
Fumigation	Highly and rapidly effective in fungi and insects, organic materials	Very toxic gases (often carcinogenic)	Kumar and Kumar, 1999; Warscheid and Braams, 2000; Salvadori, 2003
Anoxic atmosphere	Fungi are susceptible to oxygen depletion	Long exposure period, expensive equipment	Gu, 2003; Salvadori, 2003

Microbial geotechnology, i.e., microbial based technology for civil structures is an emerging discipline of science which has developed immensely in the recent years. Microbially induced carbonate precipitation has successfully emerged as a novel method to protect and remediate decayed building structures and materials. The method of use of bacteria for remediating building materials is mimicry of the nature as many carbonate rocks have been cemented by precipitation of carbonates induced by microbes. This technology of application of bacteria for precipitation of carbonates has been successfully used for solving various durability issues of different construction materials as it is novel and eco—friendly method to protect and restore the decayed construction materials (Dhami et al., 2012, 2013).

In the present review, an attempt has been made to provide an overview of the various agents responsible for deterioration of stone monuments (statues, buildings, paintings etc.) and the current methods for restoration of the important stone works with focus on microbially induced carbonate precipitation as promising technology for bioremediation of such structures. The aim is to highlight the contribution of Applied Microbiology and Biotechnology in successfully solving various problems related to durability issues of historical and important building materials.

### Deterioration of stone works: causative agents

The deterioration of historic monuments and stone works occurs due to numerous factors leading to stone dissolution, staining or color alteration, surface alterations, biocorrosion and transformations into smaller sized crystals etc. (Chand and Cameotra, 2011). In the last decades, alterations have occurred mainly due to microbial biofilm production, deposition of organic and inorganic compounds, formation of black crusts, nitratation, sulphatation and, due to residual hydrocarbons and other organic pollutants in dust (Warscheid and Braams, 2000; Fernandes, 2006; Di Pippo et al., 2009).

#### Nitrates

Nitrates, originating from the reaction of numerous oxides of nitrogen present in atmosphere (N_2_O, NO, N_2_O_3_, NO_2_, N_2_O_5_) due to pollution formed through oxidation upon reaction with water vapor produces nitrous acid and more abundant nitric acid (HNO_3_) as final products. These acids result in the formation of acid rain and attack the stone structures causing formation of calcium nitrate salts on the stone buildings (Ranalli et al., 1999).

#### Sulfates

Sulfates, which originate from oxidation of sulfur dioxide lead to the formation of sulfuric acid (H_2_SO_4_) resulting in acid rain. These acid rains cause transformation of insoluble calcium sulfate posing potential risk not only to buildings but also for humans (Ranalli et al., 1999).

#### Black crusts

Black crusts are normally formed as a result of mixing of gypsum crystals with atmospheric particles (pollen, dusts, spores, particulate matter called smog etc.) (Saiz-Jimenez, 1991; Saiz-Jimenez and Garcia del Cura, 1991). Calcium sulfate salt crusts also accumulate particles of soot originating from fossil fuel consumption and form black crusts (Kumar and Kumar, 1999; Warscheid and Braams, 2000).

#### Organic matter

Organic matter is ascribed to the lysis of microbial cells and presence of hydrocarbons originating from combustion of oil. This type of deterioration becomes more evident in the buildings located in the open as atmospheric pollution also contributes to add up the degradation process (Ranalli et al., 1999). The formation of all these crusts affect stone's texture, crystal structure, composition, coherence, water uptake and strength.

#### Microorganisms

Microorganisms (Bacteria, Archaea, Fungi, Algae, Lichens), along with mosses and higher plants, have been reported commonly on stoneworks leading to deterioration of several types of materials as stone works, wood, tapestries, papyrus, canvas, paper etc. (Cappitelli and Sorlini, 2005; McNamara and Mitchell, 2005; Ramírez et al., 2005) (Table 2). These microorganisms are amongst the major players of biodegradation of several stone work buildings. The photolithoautotrophic nature of algae and cyanobacteria facilitate colonization of stone by many other microorganisms (e.g., fungi and bacteria) (Warscheid and Braams, 2000; McNamara and Mitchell, 2005). The nitrifying bacteria (*Nitrosomonas* spp. and *Nitrobacter* spp.), capable of excreting nitrous and nitric acid and sulfur-oxidizing bacteria (*Thiobacillus* spp.) which produce sulfuric acid leads to biocorrosion of the stone material (Gómez-Alarcón et al., 1995; Warscheid and Braams, 2000). The acid formed reacts with stone constituents to produce sulfate-based crusts which upon precipitation in pores of stone cause considerable stress in porewalls. Biocorrosion also occurs by chemoorganothrophic microorganisms, including several bacteria and fungi (*Acidithiobacillus ferrooxidans*, *Bacillus* spp., *Leptospirillum* spp., *Aureobasidium* spp.) as well as lichens as they excrete organic acids. These acids have been reported to chelate the metal cations (e.g., Fe, Mg, Mn, Si, Al, Ca etc.) from minerals to form complexes which are quite stable with time (Kumar and Kumar, 1999; Warscheid and Braams, 2000; McNamara and Mitchell, 2005; Rawlings, 2005). Physical penetration by lichens and fungi also contributes to degradation. The hyphae of fungus penetrate deeply beneath the stone surface, causing not only mechanical deterioration but also transport of water and nutrients through the stone facilitating colonization of stone interior by bacteria leading to biochemical deterioration (Gómez-Alarcón and de La Torre, 1994).

**Table 2:** **Microorganisms and environmental factors involved in biodeterioration of architectural buildings and artworks (Source: Dakal and Cameotra, 2012)**.

**Microbial group**	**Microorganisms/environmental factors**	**Deterioration type**	**Mechanism**
Photoautotrophs	Cyanobacteria	Esthetic and chemical deterioration	Biofilm, color alteration, patina, crust formation, bioweathering
	Lichen	Chemical and mechanical deterioration	Extraction of nutrients from stone surface, oxalate formation, carbonic acid production, physical intrusions
	Algae	Esthetic and chemical deterioration	Biofilm, color alteration, black crusts
	Mosses and Liverworts	Esthetic and chemical deterioration	Discoloration, green gray patches, extraction of minerals
Chemoautotrophs	Sulfur oxidizing, Nitrifying bacteria	Chemical deterioration	Black custs
Chemoheterotrophs	Heterotrophic bacteria	Esthetic and chemical deterioration	Crust formation, patina, exfoliation, color alteration
	Actinomycetes	Esthetic deterioration	Whitish gray powder, patina, white salt efflorescence
	Fungi	Esthetic, chemical, physical and mechanical deterioration	Fungal diagenesis, color alteration, oxalate formation, bioweathering, physical intrusions, destabilization of stone texture
Chemoorganotrophs	Sulfur reducing bacteria	Chemical deterioration	Conversion of sulfate to sulfite
Higher plants	Higher plants	Mechanical deterioration	Intrusion of roots in cracks, pores leading to collapse and detachment of stone structure

Several attempts have been made to decrease the susceptibility to decay by many conservation treatments which includes application of surface sealing or consolidating agents to the substrate resulting in organic/inorganic precipitation of binding material in the pores of stone (Adeyemi and Gadd, 2005). These stone consolidants reestablish the binding between the grains of degraded stone. To protect the stone from water ingress and weathering agents, water repellents have also been applied. These chemicals have short efficacy due to their chemical composition and thermal expansion coefficient which are quite different from that of the stone (De Muynck et al., 2010). But due to incompatibility problems with the stone, consolidants as well as water repellents have been reported to accelerate decay of the stone material (Clifton and Frohnsdorff, 1982; Delgado Rodrigues, 2001; Moropoulou et al., 2003). Efforts have been made to introduce methods based on CaCO_3_ precipitation into the pores of limestone by few researchers. Application of saturated solution of calcium hydroxide (Lime-water technique) has been used on degraded stones so as to impart a slight water repellent and consolidating effect (Tiano et al., 1999). But, little success has been achieved till now in consolidation of stone with inorganic materials. This is because of the tendency of these materials to generate shallow and hard crusts due to their poor penetration abilities, growth of precipitated crystals, salts formation and stone particle binding ability (Clifton and Frohnsdorff, 1982).

To overcome the limitations of these conventional treatments, researchers proposed microbially induced calcium carbonate precipitation as an eco-friendly method to protect and restore degraded ornamental stones (Le Metayer-Levrel et al., 1999; Stocks-Fischer et al., 1999; Ramachandran et al., 2001; Ramakrishnan et al., 2001; De Muynck et al., 2008a,b). Although microorganisms have often been associated with detrimental effects on the integrity of stone structures, affecting mineral integrity or exacerbating powerful physical processes of deterioration (Papida et al., 2000), there is an increase of evidence that they could be used to reverse the deterioration processes on historical objects of art (Atlas et al., 1988; Lal Gauri et al., 1989b; Orial et al., 1992; Castanier et al., 1999; Perito et al., 1999; Ranalli et al., 1999).

### Microbially induced calcium carbonate precipitation

Microbially induced calcium carbonate precipitation (MICCP) is a process where an organism creates a local micro-environment, with conditions that permits precipitation of carbonates (Hamilton, 2003). Bacteria isolated from different natural habitats have been reported for their ability to precipitate calcium carbonate both in natural and laboratory conditions (Krumbein, 1979; Rodriguez-Navarro et al., 2003). Precipitation of carbonates varies based on the types of bacteria, abiotic factors such as salinity and composition of the nutrients in various environments (Knorre and Krumbein, 2000; Rivadeneyra et al., 2004). Calcium carbonate precipitation is a chemical process and influenced by four main factors such as the calcium concentration, amount of dissolved inorganic carbon (DIC), availability of nucleation sites and pH (Hammes and Verstraete, 2002). Sufficient calcium and carbonate ions are required for CaCO_3_ precipitation so that the ion activity product (IAP) exceeds the solubility constant (*K*_so_) Equations (1) and (2). From the comparison of the IAP with the *K*_so_, the saturation state (Ω) of the system can be defined; if Ω > 1, then the system is oversaturated and precipitation is likely to occur as mentioned below by Morse (1983):

(1) $$Ca^{2+}+CO_{3}^{2-}↔CaCO_{3}$$

(2) $$\begin{array}{l} \Omega =a\left(Ca^{2+}\right)a\left(CO_{3}^{2-}\right)/K_{so}\text{with}\\ \;K_{socalcite,25^{{}^{\circ}}}\;=\;4.8\times 10^{-9} \end{array}$$

As mentioned previously, the amount of carbonate ions is related to the amount of DIC and pH of a given aquatic system. However, the amount of DIC depends on several environmental parameters like temperature and partial pressure of carbon dioxide. The equilibrium reactions and constants governing the dissolution of CO_2_ in aqueous media (25°C and 1 atm) are given below in Equations (3)–(6) as suggested by Stumm and Morgan (1981):

(3) $$CO_{2}(g)↔CO_{2}(aq)\left(pK_{H}\;=\;1.468\right)$$

(4) $$CO_{2}(aq)+H_{2}O↔H_{2}CO_{3}^{*}(pK\;=\;2.84)$$

(5) $$H_{2}CO_{3}^{*}↔H^{+}\;+\;HCO_{3^{-}}(pK1\;=\;6.352)$$

(6) $$HCO_{3^{-}}↔\;CO_{3}^{2-}+\;H^{+}(pK2\;=\;10.329)$$

(7) $$\text{With }H_{2}CO_{3}^{*}\;=\;CO_{2(aq)}\;+\;H_{2}CO_{3}$$

Hammes and Verstraete (2002) suggested that microorganisms influence precipitation by altering any of the precipitation parameters described above, either separately or in various combinations with one another. MICCP has gained increasing interest in the last 20 years and found to be the primary focus of research in bio geo civil engineering because of its numerous applications.

There are mainly four groups of microorganisms involved in the process, which are: (i) photosynthetic organisms such as cyanobacteria and algae, (ii) sulfate reducing bacteria responsible for dissimilatory reduction of sulfates, (iii) organisms utilizing organic acids, and (iv) organisms that are involved in nitrogen cycle either by ammonification of amino acids/nitrate reduction or hydrolysis of urea (Stocks-Fischer et al., 1999; Hammes and Verstraete, 2002; Jargeat et al., 2003).

In aquatic environments, MICCP is primarily caused by photosynthetic organisms (McConnaughey and Whelan, 1997). Algae and cyanobacterial metabolic processes utilize dissolved CO_2_ Equation (8), which is in equilibrium with HCO^−^_3_ and CO^−^_32_ Equation (9). The removal of CO_2_ induces a shift in this equilibrium, and results in an increase in pH Equation (10) (Ehrlich, 1998) and in presence of calcium ions, this reaction leads to precipitation of calcium carbonate as mentioned by Hammes and Verstraete (2002) Equation (11).

(8) $${\text{CO}}_{2}+{\text{H}}_{2}\text{O}\to \left({\text{CH}}_{2}\text{O}\right)+{\text{O}}_{2}$$

(9) $$2\;{\text{HCO}}_{3}^{-}↔{\text{CO}}_{2}+{\text{CO}}_{3}^{2-}+{\text{H}}_{2}\text{O}$$

(10) $${\text{CO}}_{3}^{2-}+{\text{H}}_{2}\text{O}↔{\text{HCO}}_{3}^{-}+{\text{OH}}^{-}$$

(11) $${\text{Ca}}^{\text{2+}}+{\text{HCO}}_{3}^{-}+{\text{OH}}^{-}\to {\text{CaCO}}_{3}+{\text{2H}}_{2}\text{O}$$

Calcium carbonate precipitation via this pathway occurs in sea water, geological formations (Packman et al., 1999; Machel, 2001), in landfill leachates (Maliva et al., 2000) and even during the biological treatment of acid mine drainage (Kaufman et al., 1996). Chiefly, in several of the described examples for this pathway, instead of calcite, dolomite and aragonite are the predominant minerals to precipitate (Packman et al., 1999; Wright, 1999; Warthmann et al., 2000; Machel, 2001).

Calcium carbonates can also be precipitated by heterotrophic organisms, by the production of carbonate or bicarbonate and modification of the environment to favor precipitation (Castanier et al., 1999). The abiotic dissolution of gypsum (CaSO_4_.2H_2_O) Equation (12) provides an environment that is rich in both sulfate and calcium ions. In the presence of organic matter and absence of oxygen, sulfate reducing bacteria can reduce sulfate to H_2_S and release HCO^−^_3_ Equation (13) (Ehrlich, 1998; Castanier et al., 1999; Wright, 1999). If H_2_S then degasses from the environment, this results in an increase in pH and favors the precipitation of calcium carbonate Equation (11) (Castanier et al., 1999).

(12) $${\text{CaSO}}_{4\cdot }{\text{2H}}_{2}\text{O}\to {\text{Ca}}^{\text{2+}}+{\text{SO}}_{4}^{\text{2−}}+{\text{2H}}_{2}\text{O}$$

(13) $$2\left({\text{CH}}_{2}\text{O}\right)+{\text{SO}}_{4}^{2-}\to {\text{HS}}^{-}+{\text{HCO}}_{3}^{-}+{\text{CO}}_{2}+{\text{H}}_{2}\text{O}$$

Third pathway includes bacteria which use organic acids as their only source of carbon and energy wherein some common species of soil bacteria are included. Such acids include oxalate, acetate, citrate, glyoxylate, succinate and malate. The consumption of these acids results in pH increase, which leads to precipitation of carbonates in the presence of calcium ions Equations (14)–(16) (Knorre and Krumbein, 2000; Braissant et al., 2002)

(14) $${\text{CH}}_{3}{\text{COO}}^{-}+{\text{2O}}_{2}\to {\text{CO}}_{2}+{\text{H}}_{2}\text{O + }{\text{OH}}^{-}$$

(15) $${\text{2CO}}_{2}+{\text{OH}}^{-}\to {\text{CO}}_{2}+{\text{HCO}}_{3}^{-}$$

(16) $${\text{2HCO}}_{3}^{-}+{\text{Ca}}^{\text{2+}}\to {\text{CaCO}}_{3}+{\text{CO}}_{2}+{\text{H}}_{2}\text{O}$$

Numerous heterogenous bacterial groups are linked to this precipitation mechanism. Braissant et al. (2002) speculated that this pathway might be extremely common in natural environments due to the abundance of such low molecular weight acids in the soils (produced by fungi and plants).

### Microbially induced calcium carbonate precipitation via urea hydrolysis

The precipitation of carbonates by bacteria through urea hydrolysis is the most straightforward and easily controlled mechanism of MICCP with precipitation of high amounts of carbonates in less time. Stocks-Fischer et al. (1999) suggested that during microbial urease activity, 1 mol of urea is hydrolyzed intracellularly to 1 mol of ammonia and 1 mol of carbonate Equation (17), which spontaneously hydrolyzes to form additional 1 mol of ammonia and carbonic acid Equation (18). These products equilibrate in water to form bicarbonate, 1 mol of ammonium and hydroxide ions which increases the pH. The above information is mentioned below through equations as reported by Stocks-Fischer et al. (1999)

(17) $$CO{\left(NH_{2}\right)}_{2}+H_{2}O\overset{bacteria}{\to }NH_{2}COOH+NH_{3}$$

(18) $$NH_{2}COOH+H_{2}O\to NH_{3}+H_{2}CO_{3}$$

(19) $$H_{2}CO_{3}\to 2H^{+}+2CO_{3}^{2-}$$

(20) $$NH_{3}+H_{2}O\to NH^{4+}+OH^{-}$$

(21) $$Ca^{2+}+CO_{3}^{2-}\to CaCO_{3}(K_{SP}=3.8\times 10^{-9})$$

*K_SP_* is the solubility product in Equation (21).

**Figure 1:**
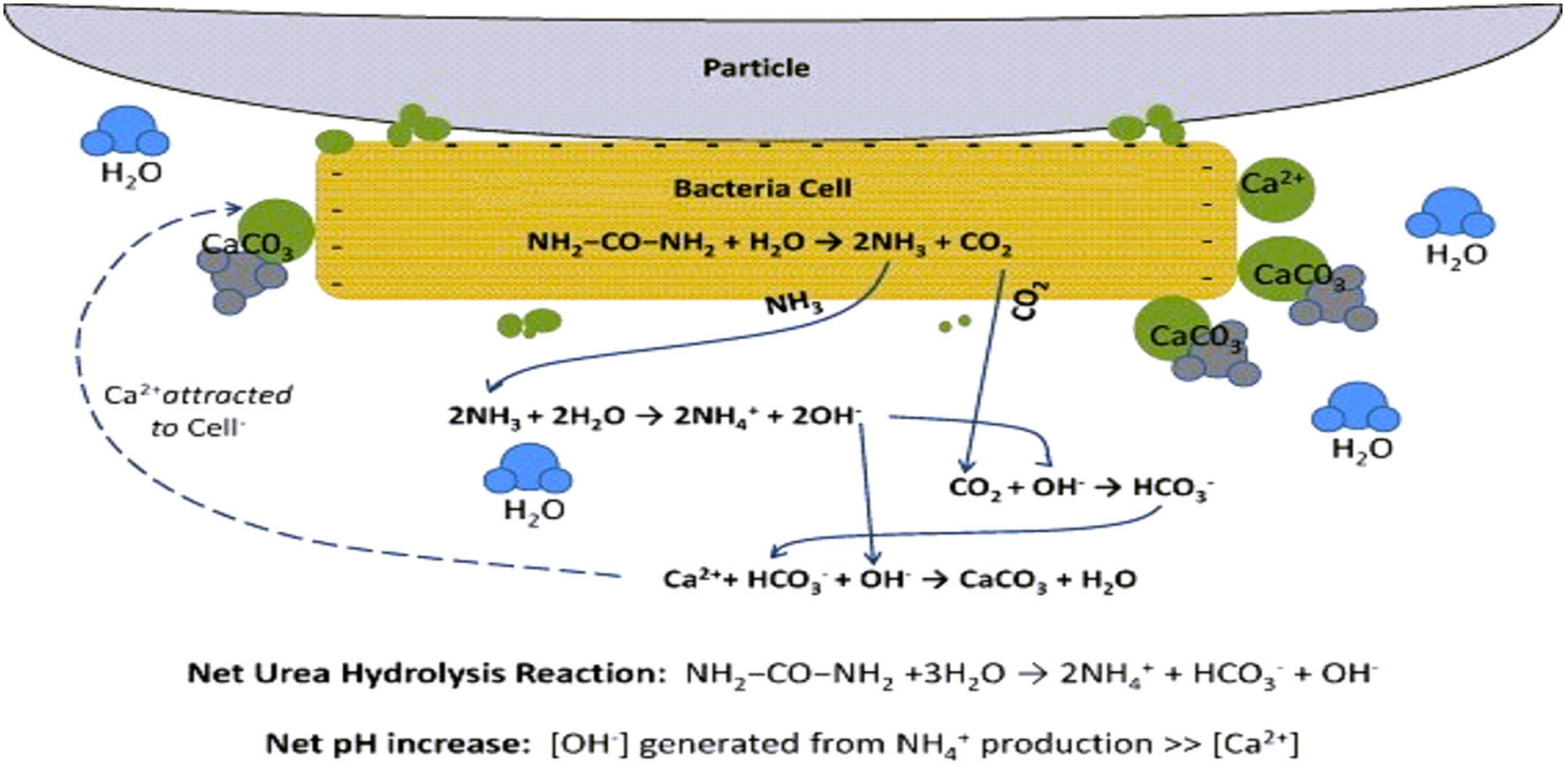
**Bacteria serving as nucleation site for CaCO_3_ precipitation in the substrate particles**. Calcium ions in the solution are attracted to the bacterial cell wall due to its negative charge. When urea is added to bacteria, dissolved inorganic carbon (DIC) and ammonium (AMM) are released in the microenvironment of the bacteria. In the presence of calcium ions, this leads to local supersaturation and finally there is precipitation of calcium carbonates which act as binder between loose substrate particles (Source: DeJong et al., 2010).

The main role of bacteria has been ascribed to their ability to create an alkaline environment through various physiological activities. The surface of bacteria plays an important role in precipitation of calcium (Fortin et al., 1997). At a neutral pH, the metal ions which are positively charged get bound on the surfaces bacteria due to presence of several negatively charged groups which favors heterogenous nucleation (Douglas and Beveridge, 1998; Bäuerlein, 2003). Generally, the precipitation of carbonates on the external surface of bacterial cells occurs by successive stratification (Pentecost and Bauld, 1988; Castanier et al., 1999) and these bacterial cells get embedded in growing carbonate crystals (Rivadeneyra et al., 1998) (Figure 1).

Biochemical reactions which take place in the urea-CaCl_2_ medium leading to precipitation of CaCO_3_ at the cell surface (Stocks-Fischer et al., 1999) and act as binders in between the substrate particles can be listed as:

(22) $$Ca^{2+}+Cell\to Cell-Ca^{2+}$$

(23) $$Cl^{-}+HCO^{3-}+NH_{3}\to NH_{4}Cl+CO_{3}^{2-}$$

(24) $$Cell-Ca^{2+}+CO_{3}^{2-}\to Cell-CaCO_{3}$$

So, mineralizing activities of microorganisms can now be harnessed positively, making them essential to the existence of ecology of Earth. The use of microbially induced carbonate biominerals is becoming increasingly popular day by day. From removal of heavy metals and radio nucleotides, removal of calcium from wastewater and biodegradation of pollutants, atmospheric CO_2_ sequestration, modifying the properties of soil and filler in rubber and plastics to fluorescent markers in stationery ink and remediation of building materials, bacterial carbonates are serving many fields (Dhami et al., 2013).

### Microbial carbonates: remediation of limestone

Boquet et al. (1973) firstly demonstrated the precipitation of calcium carbonate by soil bacteria under laboratory conditions. Previous researchers showed precipitation of carbonates by marine bacteria only in liquid media while Drew (1911) and Shinano (1972), investigated the carbonate precipitation by soil bacteria on solid media and obtained best results with B4 medium. Among the organisms tested, several *Bacillus* strains and *Pseudomonas aeruginosa* were observed to form crystals. Castanier et al. (1999) reported the microbial origin of limestone while Adolphe et al. (1989) further demonstrated the bacterial origin of the calcite crusts and found great resistance against erosion by this calcite layer. Adolphe et al. (1990) applied patent for the treatment of artificial surfaces by surface coatings produced by microorganisms and formed a company “Calcite Bioconcept.” The promising results of “Calcite Bioconcept” encouraged many researchers to look for different approaches for bioremediation of stone by microbial carbonates. First approach was based on usage of different microbes and metabolic pathways or delivery systems to overcome limitations of “Calcite Bioconcept” technique while in second approach, no microbes were applied directly rather inducing macromolecules along with supersaturated solution of calcium carbonate and carbonate precipitation by microbiota inhabiting the stone were investigated.

The carbonate precipitation ability of bacteria had been demonstrated under laboratory but further experiments are required to assess the viability and carbonate precipitation ability of these bacteria *in situ*. The collaboration between the University of Nantes, the Laboratory for the research of historic monuments (LRMH) and the “Calcite Bioconcept” (Le Metayer-Levrel et al., 1999) led to the optimization and industrialization of this concept. Upon investigating different bacteria, Castanier et al. (1999) reported the highest performance by *B. cereus* which was further selected for *in situ* applications (Orial, 2000). This paved way to optimization of the nutrient media with source of proteins for the oxidative deamination of amino acids by aerobiosis and nitrogen source for the dissimilatory reduction of nitrate in anaerobiosis and microaerophilic conditions along with a fungicide to prevent undesirable growth of fungi on the stone (Orial et al., 2002). The first application *in situ* was carried out in 1993 in Thouars on the tower of the Saint Médard Church. It was reported that presence of the biocalcin reduced water absorption rate to five time s and did not affect the esthetic appearance (Le Metayer-Levrel et al., 1999). But Orial (2000) suggested that after every 10 years a new biocalcin treatment was needed to restore its protective effect. This technology was also applied on limestone statuaries in different climatic environments where it was found to be highly successful even after 4 years of application. Addition of natural pigments into the nutritional medium created a surficial patina with the biodeposition treatment. The pigments integrated into the biocalcin resulted a persistent light coloring to the stone. This technique concealed some newly replaced stones on a monument (Le Metayer-Levrel et al., 1999). Alhough *B. cereus* was quite effective in bioconsolidation, but the layer of new cement induced was very thin, just a few microns—thick. Formation of endospores and formation of uncontrolled biofilm by *Bacillus* species provides a drawback in stone conservation. Hence, Rodriguez-Navarro et al. (2003) proposed the use of *Myxococcus Xanthus*, which is Gram-negative, nonpathogenic soil bacteria. This bacterium is known to induce the precipitation of carbonates, sulfates and phosphates in wide range of solid and liquid media (González-Muñoz et al., 1993, 1996; Ben Omar et al., 1995, 1998; Ben Chekroun et al., 2004; Rodriguez-Navarro et al., 2007). Application of this bacterial suspension on stone specimens showed no fruiting bodies and no uncontrolled bacterial growth. Calcium carbonate precipitation was observed up to a depth of several 100 μm (>500 μm) without plugging or blocking of the pores. Plugging mainly occurs due to biofilm formation through extracellular polymeric substance (EPS) (Tiano et al., 1999).

**Figure 2:**
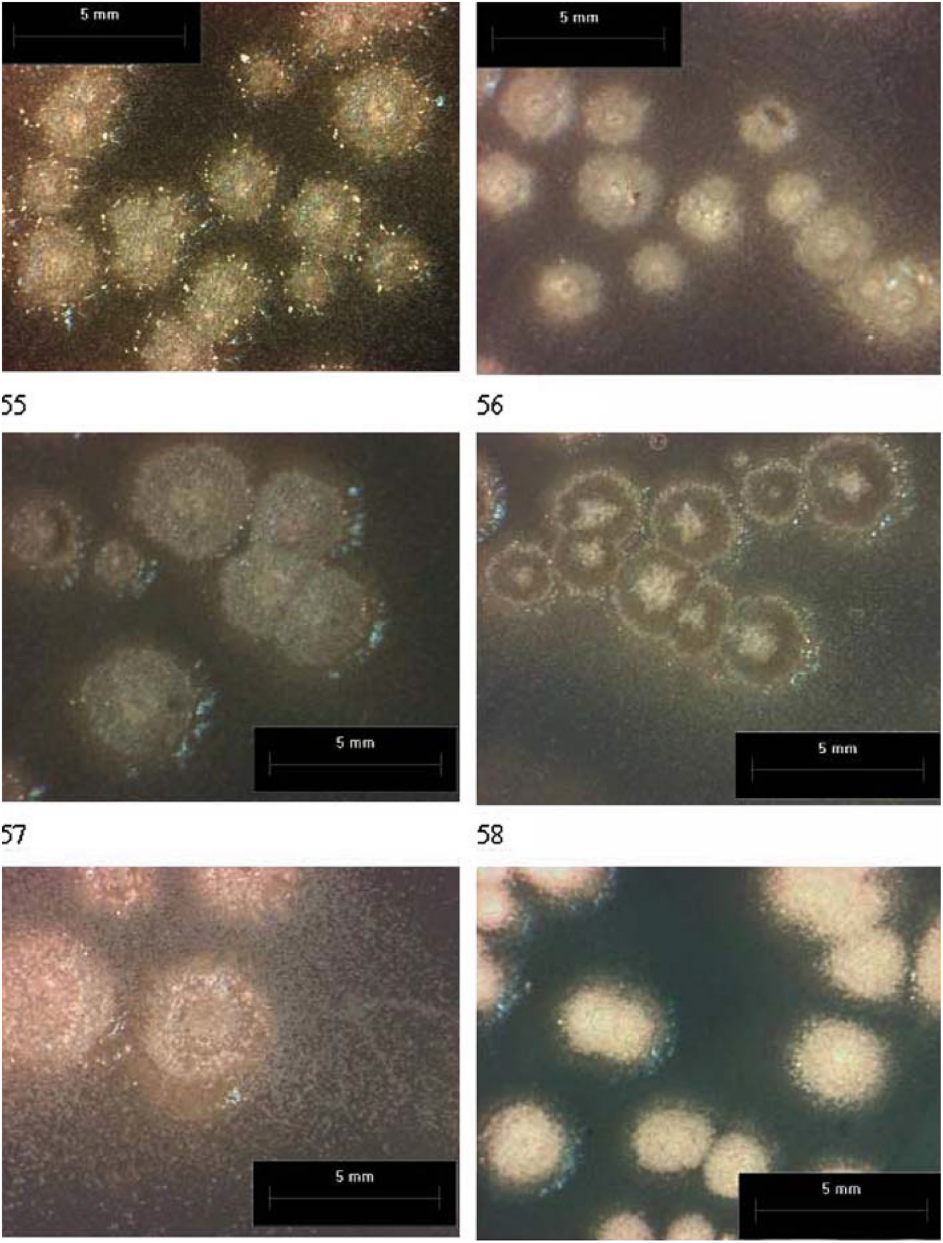
**Colonies of 6 different strains of *B. sphaericus* and *B. lentus* on agar plates and their ability to encrust themselves in calcium carbonate (Source: Dick et al., 2006)**.

Tiano et al. (1999) studied the effect of microbial calcite crystals on Pietra di Lecce bioclastic limestone by *Micrococcus* spp. and *Bacillus subtilis* and their results showed a significant reduction in water absorption. The authors also commented some negative consequences, such as (i) the formation of new products due to chemical reactions between stone minerals and some by-products originating from the metabolism of bacteria, and (ii) the formation of stained patches because of the growth of air-borne fungi. To avoid such short comings, the authors used some natural and synthetic polypeptides to control the calcite crystal growth in the pores. Use of organic matrix macromolecules (OMM) isolated from *Mytilus californianus* shells was proposed to induce the precipitation of calcium carbonate within the pores of the stone (Tiano et al., 1992; Tiano, 1995). Slight decrease in porosity and water absorption by capillarity was observed in this case (Tiano, 1995). This method was not much beneficial due to the complexity of isolation procedure as well as less yield of usable product (Tiano et al., 1999). Hence, in place of this bio inducing macromolecules (BIM) rich in aspartic acid groups, Tiano et al. (2006) proposed to use acid functionalized proteins such as polyaspartic acid. Calcium and carbonate ions were supplied for calcite crystal growth, by addition of ammonium carbonate and calcium chloride solution or a solution of saturated bicarbonate. The consolidating effect was observed to be very low compared to ethylsilicates (Tiano et al., 2006). Dick et al. (2006) observed 50% reduction in water absorption by treating limestone cubes with two strains of *B. sphaericus* (Figure 2).

**Figure 3:**
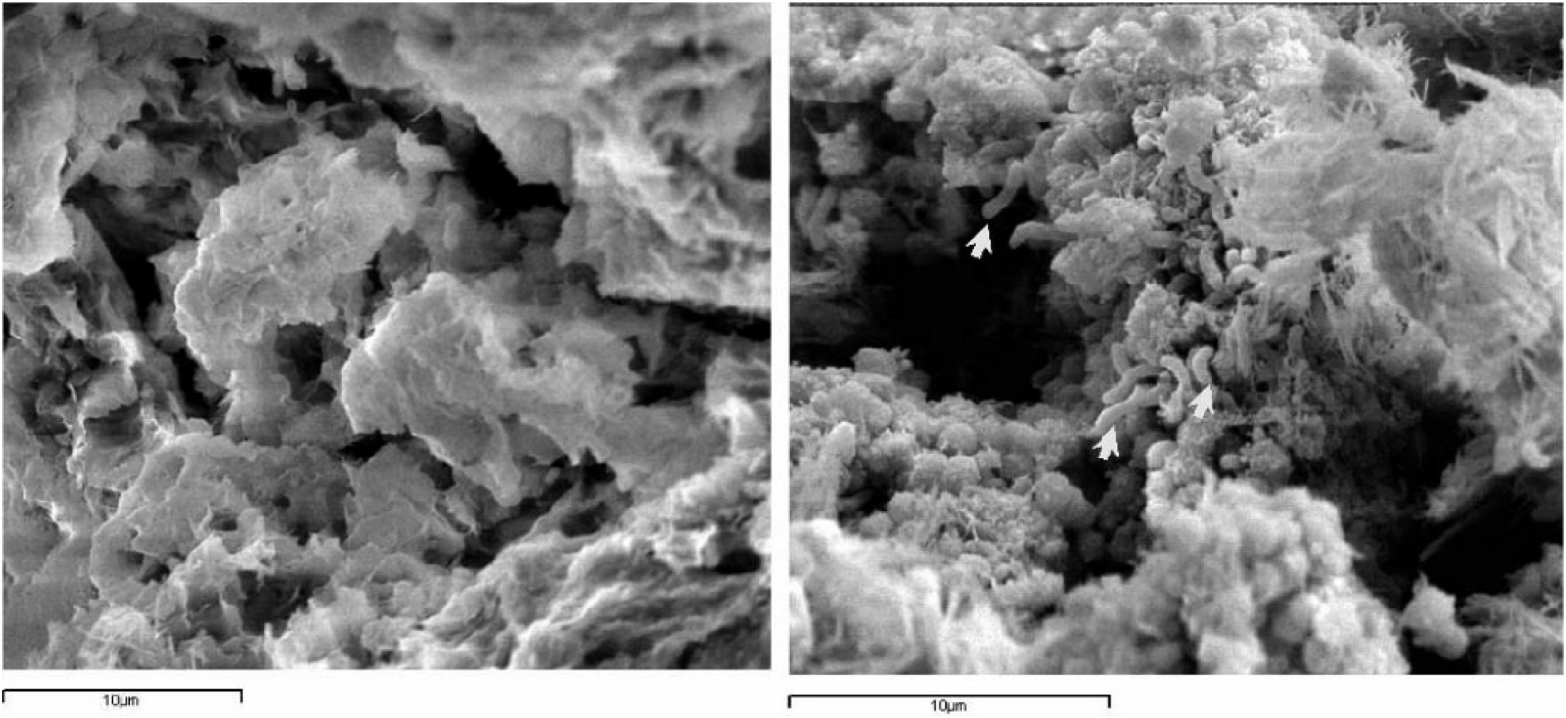
**Scanning electron microscopy (SEM) observations of Carbogel without (left) and with (right) *D. vulgaris* subsp*. vulgaris* ATCC 29579 cells (Source: Cappitelli et al., 2006)**.

To improve the methodologies for delivering bacterial cells to stone surfaces and also to control the side effects of bacteria to the stone, various carrier materials were looked upon. Ranalli et al. (1997) used sepiolite for delivering *Desulfovibrio vulgaris* and *D. desulfuricans*, as it provides anaerobic conditions, humidity and shorten the treatment time. Cappitelli et al. (2006) proposed Carbogel as a delivery system for bacteria due to its high retention of viable bacteria and less time to entrap cells (Figure 3). Different methodologies where microbial calcite has been deposited as a layer on surface of stone is presented in Table 3.

**Table 3:** **Overview of different methodologies where microbial calcite has been deposited as a layer on surface of stone**.

**Experimental methods**	**Application procedure**			**Authors**
**Inoculum**	**Bacteria**	**Nutrients**	**Evaluation procedures**	
Culture in exponential phase: 10^7^–10^9^ cells/ml	Spraying	Spraying (5 times)	Water absorption, SEM analysis, surface roughness, colorimetery and plate count	Calcite bioconcept Le Metayer-Levrel et al., 1999
Overnight culture 10^6^ cells cm^−2^	Brushing on water saturated specimens	Wetting every day for 15 days	Water absorption, colorimeteric measurements, stone cohesion	Tiano et al., 1999
2% inoculums	Immersion in growing bacterial culture (shaking or stationary conditions) for 30 days		Stone cohesion, weight increase, XRD and SEM analysis, porosimetery analysis	Rodriguez-Navarro et al., 2003
1% inoculums	Immersion in growing bacterial culture (intermediate wetting) for 28 days		Water absorption, SEM analysis	Dick et al., 2006
10^8^ cells ml^−1^	Spraying	In Carbogel	Water absorption and drying due to evaporation	May, 2005
n.d.^*^	n.d.^*^	Immersion in test solution or spraying (*in situ*) tests	Water absorption, colorimeteric measurements, stone cohesion, staining of newly formed calcite with Alizarin Red S and Calcein	Tiano et al., 2006
Overnight culture 10^7^ – 10^9^ cells ml^−1^	Immersion for 1 day	Immersion for 4 days	Weight increase, water absorption, gas permeability, chloride migration, carbonation, freezing and thawing, SEM and XRD analysis	De Muynck et al., 2008a

* *n.d., not defined*.

Precipitation of calcite crystals by fresh water bacteria on limestone significantly reduced the pore sizes of the stone (Zamarreno et al., 2009). Calcite crystals were deposited around and inside open pore spaces. Application of calcite crystals filled 43–49% of the open pore spaces which was 20% higher than the application of the medium alone. De Muynck et al. (2012) reported *B. sphaericus* to be very efficient strain for consolidation of limestone specimens at range of temperatures (10, 20, 28, 37°C). This isolate led to 64% lower weight loss upon sonication and 46% decreased sorptivity in treated limestone specimens compared to the control specimens. De Muynck et al. (2011) recently applied bacterial calcite in two types of stones: microporous and macroporous. They reported that application of bacterial carbonates is more successful in macroporous stone where it occurs to a larger extent and at greater depths than in microporous stone. It has also been shown on laboratory scale that several bacterial strains (such as *Pseudomonas stutzeri, P. aeruginosa, D. vulgaris*, and *D. desulfuricans*) are not only able to denitrify and desulfuricate harmful masonry salts such as nitrate and sulfate (Lal Gauri et al., 1989a; Heselmeyer et al., 1991; Ranalli et al., 1999) but also mineralize organic residues or pollutants like carbohydrates, waxes or hydrocarbons that commonly occur in crusts on stonework (Warscheid et al., 1991; Saiz-Jimenez, 1997; Ranalli et al., 1999).

From the above mentioned applications, microbial concrete seems to bring a new revolution in the civil industry. Use of bacteria to improve the durability of building materials has drawn the attention of research groups all over the world. But several challenges have to be met before acceptance of this technology by conservators.

## Limitations and challenges

Though there are many advantages of MICCP technology for bioremediation of several stone structures but there are a few limitations also. In comparison to chemical treatments, biobased treatments are found to be more complex because the microbial activity depends on many environmental factors such as temperature, pH, concentrations of donors and acceptors of electrons, concentrations and diffusion rates of nutrients and metabolites. Design of experiments for biodeposition treatments require a huge data of the biological processes (growth, biosynthesis, specific enzymatic activities), chemical reactions accompanied with formation of insoluble compounds, physic—chemical processes as precipitation, crystallization, and adhesion.

Due to this complexity, its usage at large-scale has not been so encouraging. The inconvenient application procedures also are major gaps for successful commercialization. The precipitation of carbonates mainly depends on time required for carbonate formation. If precipitation time increases, then the amounts of EPS production increases, and hence plugging but multiple applications of nutrients and usage of carrier materials have significant influence on the total cost of treatment (Le Metayer-Levrel et al., 1999; May, 2005). Production of ammonia during hydrolysis of urea poses environmental as well as leads to discoloration of stone (Sutton et al., 2008; Tobler et al., 2011). Ammonium is also converted to nitric acid due to the action of denitrifying bacteria which results in significant damage to the stone. Additional research is necessary to overcome this problem. As the amounts of carbonate precipitates formed are dependent on amount of calcium added, increased concentration of calcium leads to accumulation of salts and paves way to efflorescence and damage to crystallization. The survival of bacteria within the stone material also influences the extent of calcification. As the laboratory grade nutrient media limit the economical usage of this technology for commercial scales, there is great need to look for alternative economical and cheap medium ingredients as corn steep liquor and lactose mother liquor (Achal et al., 2009, 2010). Large scale production of bacterial cultures is also a hindrance in the path of success of this technology over traditional treatments. The above mentioned concerns limit the use of MICCP for practical applications in various fields in comparison to the traditional methods.

## Conclusion

Microbially induced calcium carbonate precipitation technology has been found to be highly promising with potential to successfully remediate and protect several stone structures. The eco–friendly, self-healing and highly durable nature of these bio-binders encourage their biotechnological applications for several purposes. Carbonate formation by this technology has been found to be very easy and convenient. The potential of these bio-binders has brought a new revolution in field of civil engineering but still there has been much to explore in order to bring this environmentally safe, cost effective and convenient technology from lab to field scales. There is need to assess the long term efficacy of microbial carbonates and compared to chemical binders. As the success of this technology needs experts from varying sectors from Microbiologists to Geologists to Civil Engineers, researchers from all around the globe should work together to make this multi-disciplinary research move toward commercial scale applications at a higher pace.

### Conflict of interest statement

The authors declare that the research was conducted in the absence of any commercial or financial relationships that could be construed as a potential conflict of interest.
